# A Non-Contact Phosphor Thermometry Technique for Determining the Optical Absorptivity of Materials

**DOI:** 10.3390/ma18204806

**Published:** 2025-10-21

**Authors:** Thomas M. F. Hutchinson, Matthew Davies, Callum Fisk, Hazem Zied, Jon R. Willmott, Matthew J. Hobbs

**Affiliations:** Sensor Systems Group, Semiconductor and Quantum Technologies, School of Electrical & Electronic Engineering, The University of Sheffield, Portobello Centre, Pitt Street, Sheffield S1 4ET, UK; mmjdavies1@sheffield.ac.uk (M.D.); cfisk1@sheffield.ac.uk (C.F.); hkmzied1@sheffield.ac.uk (H.Z.); j.r.willmott@sheffield.ac.uk (J.R.W.)

**Keywords:** absorptivity, metrology, phosphor, photoluminescence, thermometry

## Abstract

This work presents a bespoke, non-contact, and low-cost Phosphor Thermometry (PT) technique for the measurement of material absorptivity. The approach circumvents the challenges associated with traditional and intrusive calorimetric techniques, which require secure contact with the sample substrate. A thermographic phosphor (TP), Manganese-activated Magnesium Fluorogermanate (MFG), was used as a two-colour thermometer utilising the peak intensity ratio technique, enabling an empirical temperature measurement of a given Material Under Test (MUT). The system was calibrated to temperature across a dynamic range of 20°C to 140°C and subsequently assessed in terms of noise and relative sensitivity. A mathematical model describing the thermal behaviour of the samples was subsequently developed and used to infer the absorptivity value of the MUTs. Two paints, Black 3.0^®^ and Avian-B500^®^, with known but contrasting absorptivities, were analysed, resulting in measured absorptivity values of 0.9385 and 0.0651 within a range of 0.0081 and 0.0127 for the two paints, respectively. Subsequent mixtures of both paints, with inherent unknown absorptivities, provided resolvable and incremental steps between the two extremities. Further measurements at specific narrow-band wavelengths of 600nm and 1550nm of Black 3.0^®^ were performed, yielding median absorptivity values of 0.9598 and 0.9172 within a range of 0.0168 and 0.0396, respectively, therefore demonstrating the technique for the measurement of material absorptivity at discrete wavelengths. The potential of a non-contact calorimetric PT technique could provide a scalable, non-intrusive, and low-cost solution for measuring the wavelength-dependent absorptivity values of materials that are used across engineering and research fields.

## 1. Introduction

The spectral absorptivity of a material can be defined as the proportion of incident Electromagnetic Radiation (EMR) absorbed and subsequently converted into thermal energy by that material as a function of wavelength [1]. Wavelength-dependent absorptivity is a critical thermo-physical property that influences the efficiency of various thermal systems. Accurate knowledge of this absorptivity, even if at only a limited number of discrete points, is fundamental for optimising the design of such systems [2]. For instance, additive manufacturing techniques such as Selective Laser Melting (SLM) require a stable melt pool during the processing of metal powders; this is crucial for ensuring quality within the manufactured parts [3]. The stability of the melt pool is dictated by the wavelength-dependent absorptivity of the material being processed, making it a highly desirable parameter. However, this absorptivity is often unknown or poorly characterised [4]. Therefore, the typical 1064nm wavelength laser used within SLM systems is not necessarily the optimal wavelength for the material being processed [5]. For example, the optimal absorption wavelength for aluminium occurs at 808nm [6], while copper absorbs most efficiently within the Ultraviolet (UV) spectral region [7]. Having an incomplete understanding of a specific material’s absorptivity can impact upon part quality, leading to defects, distortions, and excess energy consumption during processing [8]. Similarly, materials used within space exploration applications have inherent wavelength-dependent properties, which, in the vacuum of space, are perpetually bombarded with EMR. The degree of radiation that they absorb directly dictates their temperature, which can compromise the function of sensitive equipment, particularly systems that must remain at cryogenic temperatures [9,10]. Further examples include solar absorbers [11], radiative cooling surfaces [12], thermal coatings [13], and measurement uncertainty within non-contact temperature measurements [14]. Such applications all have unique absorptivity signatures that are ultimately impacted by wavelength-dependent properties.

Two genres of measurement technique exist for measuring the absorptivity of a material: radiometric [15] and calorimetric [9]. Radiometric methods are typically used to measure emitted and/or reflected Infrared (IR) from an object, using IR imagers [16,17]. This measurement technique is limited by the requirement of knowing the target object’s independent emissivity, temperature, and the reflection of ambient radiation, which can be difficult to isolate without significant calibration and corrections [18]. A subset of radiometric techniques includes hyperspectral methods, which have been demonstrated to determine material absorptivity across a wide range of wavelengths [19]. These systems operate within the ranges of UV to Very Long Wave Infrared (VLWIR), becoming increasingly expensive as you move from the visible spectrum into the IR spectrum [20,21]. In general, radiometric systems are bulky, expensive, and time-intensive and require complex calibrations. In contrast, calorimetric methods determine a material’s absorptivity through measuring the thermal energy either lost or gained by a sample over time [9,22]. This approach typically involves the deployment of multiple thermocouples at different positions across the sample surface and is used to measure the mean temperature fluctuation within metal powders and, therefore, enable their absorptivity to be inferred [23]. However, the use of thermocouples is limited by their slow response times and the difficulty in achieving the reliable thermal contact required for accurate measurements [24,25].

Among the various alternative temperature measurement techniques that are emerging, Phosphor Thermometry (PT) is a promising, semi-invasive technique for measuring surface temperatures with high precision and reliability [26,27]. Research outputs investigating novel Photoluminecent (PL) materials and their metrological applications has grown significantly over the last decade, highlighting the growing importance of this technology [28]. PT has been successfully demonstrated in fields such as combustion diagnostics [29], aerospace thermal management [30], and nuclear safety [31]. The emissivity independence and ability to negate changes within the background radiation of PT places it in a position to overcome the aforementioned traditional thermometry techniques [31]. PT involves the use of Thermographic Phosphors (TPs), which have temperature-dependent spectral emission properties [32]. The most common approaches for TP temperature calibration and measurement can be categorised into three main methods: decay rate [33], rise time [34], or the intensity ratio [35]. Numerous studies on TPs have been conducted for the application within PT, many doped with rare earth ions, such as Erbium ($Er^{3+}$), or transition metals such as Manganese (Mn) [36,37,38]. Of all the various TPs available, the most dominant and extensively studied TP within the domain of temperature measurements is Manganese-activated Magnesium Fluorogermanate (MFG) ($Mg_{4}FGeO_{6}$:Mn) [39,40]. The MFG used within this work was purchased from Phosphor Technology Inc. (Phosphor Technology Ltd., Stevenage, Hertfordshire, UK) with product code EQD25/F-U1 [41]. MFG operates via down-conversion, which emits visible light when stimulated by an external light source with a shorter wavelength output, such as UV Light Emitting Diodes (LEDs) or lasers [42,43]. MFG has two distinct spectral emission regions, with peaks at 630nm and 660nm. These two peaks demonstrate clear but opposing thermal behaviour, with the 630nm peak intensity increasing and the 660nm peak decreasing in response to an increase in temperature. The predictable thermal response of MFG allows for precise calibration against temperature by taking the ratio of the spectral emission energy [40]. Changes in the temperature of a surface can be monitored by adhering the TP to the target object and measuring its temperature-dependent light emission using radiometry; the amount of energy absorbed by that object is directly linked to its temperature [27].

Utilisation of PT as a calorimetric technique to measure material absorptivity offers distinct advantages over existing calorimetric methods. Firstly, unlike thermocouples, PT is a semi-invasive method, which allows for measurement of thermal changes without the need for physical contact [44]. Therefore, it is applicable to a wide range of surface finishes, including rough, coated, metallic materials. Secondly, PT provides high sensitivity and spatial resolution to enable real-time measurements within dynamic or harsh environments, including cryogenic conditions [31]. In contrast, thermocouples cannot accurately track rapid temperature changes due to their slow response times, which are governed by the need to reach thermal equilibrium [24]. Furthermore, PT decouples the temperature-dependent luminescent signal from the underlying radiative properties of the Material Under Test (MUT), enabling accurate absorptivity measurements for materials that have complex or unknown emissivity characteristics [45,46,47]. Finally, combining PT with low-cost optical components and Silicon Photodiode-based Radiometers (SiRs) enables the development of affordable and portable absorptivity measurement systems that can alleviate the drawbacks associated with traditional absorptivity characterisation methods. PT, therefore, has the potential to enable extension of absorptivity measurements beyond the traditional confines of specialist laboratories.

This study introduces a novel PT technique, incorporating mathematical modelling, to measure material absorptivity. The technique was validated by measuring the absorptivity of paints with known, but contrasting, absorptivity values. Subsequent mixtures of these paints were curated with unknown, but predictable, absorptivities values, which were measured. The approach involved applying MFG and the MUT to opposing sides of a shared substrate. Each sample was heated using a blackbody (thermal source), and the MUT’s absorptivity was inferred using a combination of direct calorimetry and mathematical modelling (Equation (3)) [48]. The model was fitted to the experimental temperature data within the range temperature of 20 C to 140 C by iteratively adjusting the absorptivity value until the theoretical and measured temperature gradients aligned within 95% confidence bounds of a linear fit to the experimental data. Our approach effectively circumvents the limitations of traditional calorimetry and radiometry methods, providing a non-contact, practical, and scalable solution with significant promise for a diverse range of thermal efficiency engineering challenges.

## 2. Materials and Methods

### 2.1. Instrument Design Overview

**Figure 1:**
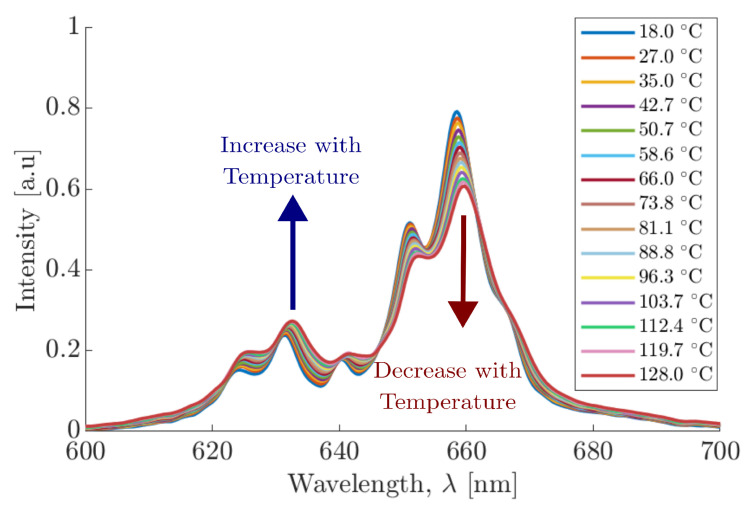
Emission spectra of MFG at different temperatures within the spectral wavelength range of 600nm to 700nm.

TPs are inorganic materials that consist of a host lattice doped with rare-earth ions [49] or transition metals [50,51,52]. They have an inherent temperature dependence that affects the intensity of their PL emissions; this makes them ideally suited for determining surface temperature [26]. MFG was chosen as the TP in this work for its wide availability, well-understood thermal properties, and distinct dual spectral peaks circa 630nm and 660nm. The dual emission peaks exhibited by MFG, as a function of wavelength and temperature, are shown in Figure 1. These emission peaks, and their surrounding spectral regions, increase and decrease as a function of temperature, respectively, and are well defined at an isosbestic point of 645nm ± 5. Isolating the spectral regions with optical filters and measuring their emission changes with temperature allows for the development of a two-colour ratio thermometer [53,54,55]. Unlike measurement of a single emission peak, the intensity ratio method boasts reduced sensitivity to variations in excitation intensity. This negates fluctuations within the excitation source since the intensity ratio is a relative measurement as opposed to absolute. Furthermore, MFG has a temperature-sensitivity range between room temperature and approximately 700°C, making it suitable for the temperature ranges investigated within this work [29].

**Figure 2:**
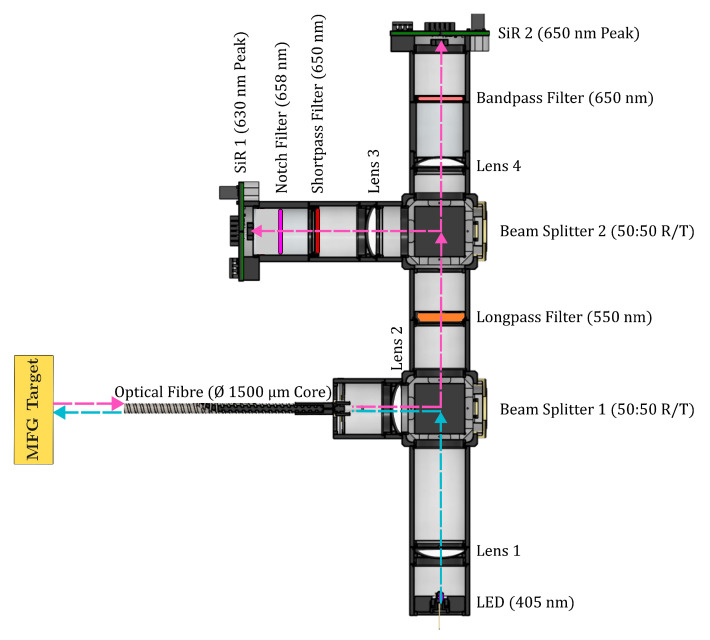
Instrument design. Top-down, cross-section. The MFG emissions are isolated by the optical filters. SiR 1 receives a peak wavelength of 630nm, and SiR 2 receives a peak wavelength of 650nm. The blue arrows indicate the path of the incident UV LED. The pink arrows indicate the path of the MFG emission after UV excitation.

A top-down cross-section of the PT setup is illustrated in Figure 2. The PT design utilised a 405nm LED (Thorlabs Inc., Newton, NJ, USA) to induce excitation of the MFG. The MFG emissions were coupled and isolated using optical filters, N-BK7 plano-convex lenses (focal length, f=50mm) (Thorlabs Inc., Newton, NJ, USA), and beam splitters (50:50 R/T) (Thorlabs Inc., Newton, NJ, USA) to isolate and direct the signal towards two SiRs. A full bill of materials can be found in Table A1.

The light emitted from the LED was first collimated by Lens 1, onto Beam Splitter 1 (50:50 R/T), which directed the emissions towards Lens 2. Lens 2 subsequently focused the LED light through a 1500µm diameter core Optical Fibre (Thorlabs Inc., Newton, NJ, USA), irradiating the MFG target. The resulting MFG emissions were emitted back through the Optical Fibre, returning to Beam Splitter 1, directing the resultant signal though a 550nm longpass filter (Thorlabs Inc., Newton, NJ, USA), removing the background emissions coming from the LED. Upon the signal reaching Beam Splitter 2 (50:50 R/T), the isolated MFG signal was directed toward the two SiRs channels with focusing Lenses 2 and 4.

To determine the optical filters needed to optimise the PT calibration and resolution, spectral data of the MFG at various temperatures were measured using a CCS200—Compact Spectrometer (Thorlabs Inc., Newton, NJ, USA), as shown in Figure 1 [56]. The spectrometer was located after the 550nm long pass filter, via a port for an optical fibre, negating the use of any subsequent materials from and including Beam Splitter 2. Optical filter data was supplied by the manufacturer and applied to the MFG spectral data; this allowed for the development of a calibration model to inform the ideal combination of optical filters needed to optimise the temperature resolution of the PT. Finally, each SiR was coupled with specific optical filters, designed to isolate the two distinct MFG spectral emission regions:

- SiR 1 (630nm peak): Coupled with a 650nm shortpass filter (Thorlabs Inc., Newton, NJ, USA) and 658±26nm notch filter (Thorlabs Inc., Newton, NJ, USA) to isolate region 1, with a peak intensity at 630nm, which increases proportionally to increased temperature. The 650nm filter was used to select the correct region, while the 658nm notch filter provided a sharp rejection of the neighbouring emissions.
- SiR 2 (650nm peak): Coupled with a 650±10nm bandpass filter (Thorlabs Inc., Newton, NJ, USA) to isolate region 2, which decreases inversely proportional to increased temperature. The 650nm bandpass filter was chosen to reject the influence of the spectral broadening; this was observed when using bandpass filters close to a 660nm peak, causing an increase in the total signal intensity in that region, as opposed to the expected decrease.

**Figure 3:**
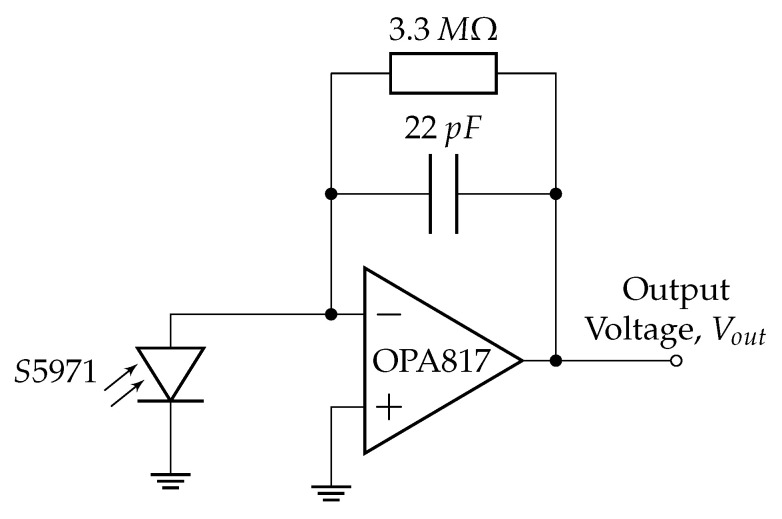
Single-stage amplifier circuit diagram for radiometers.

The basic SiR circuit design is shown in Figure 3. The circuit amplifies and converts to a voltage the photocurrent generated by an S5971 Silicon Photodiode (SiPD) (Hamamatsu Photonics K.K., Hamamatsu, Japan) in response to the incident MFG photoluminescent emission [57]. The SiPDs feature a 1.1mm^2^ active area and a nominal wavelength range of 320nm to 1060nm with a peak sensitivity of 0.64A/W at 900nm [57]. The circuit consists of a single-stage Transimpedance Amplifier (TIA) based on the OPA817 operational amplifier (Texas Instruments, Dallas, TX, USA). OPA817s were selected for their low noise performance and high gain bandwidth product, making them suitable for weak signal detection and alleviating some uncertainty in the Signal-to-Noise Ratio (SNR).

The SiRs were connected to a National Instruments FlexLogger DAQ unit (National Instruments, Austin, TX, USA), capable of capturing the analogue output voltages from the SiRs. The SiRs detected the signal changes from the isolated spectral regions of the MFG emissions, allowing the distinct PL intensities, $I_{n}\left(\lambda \right)$, to be measured as a function of temperature. By analysing the output from the two isolated spectral regions at different temperatures, the change in ratio, ΔR(T), between the two signals was used to infer the temperature of the sample’s surface. The calibration gradient, *m*, is equivalent to the ratio change-per-degree (dR/dT) and represents the resolution of the PT, where a larger gradient indicates greater sensitivity, translating small temperature fluctuations into more significant changes in the measured signal ratio. Consequently, larger calibration gradients allow the PT to resolve finer temperature increments, thereby enhancing the overall measurement resolution.

Measurement of the response time of the SiRs was performed using an Agilent Technologies Inc. 33210A waveform generator (Agilent Technologies Inc., Santa Clara, CA, USA), which supplied a 10kHz square wave to a 405nm LED. The sample rate was chosen based on the 10–90% rise time, which was found to be 100 µs. For reference, the decay rate of MFG phosphorescence is in the region of 3.5–4 ms at circa room temperature [31]. The changes in the aforementioned decay rate and rise time, as a function of temperature, are common PL temperature calibration techniques and are often used when the intensity ratio is not applicable [33,34].

To calibrate the PT against temperature, the ratio of the two SiR signals was taken, as per Equation (1). The PT optical fibre was positioned at a distance of 50mm from an MFG-coated calibration disc (outlined in Section 2.2) and placed onto a hot plate with the MFG facing upwards. A thermocouple was mounted inside a 12.7mm deep blind hole, using a silver-based thermal epoxy [58], and used to provide a valid and well-characterised temperature reference. The PT SiRs outputs were recorded by the DAQ unit with an acquisition time of 100 µs, which was later filtered using a 1 s integration time. For each temperature set point, 60 s of data was taken, starting at room temperature. The hot-plate temperature was increased in increments of 10°C, dwelling at each set point for 30 min to ensure thermal equilibrium. The process was systematically repeated until a top temperature of 140°C was reached.

(1) $$R\left(T\right)=\frac{I(\lambda_{630\;nm})}{I(\lambda_{650\;nm})}$$

The tolerance within a commercially available K-type thermocouple, as specified in BS EN 60584-2:1993, can often exceed ±1.5°C [59]. Therefore, an acceptable Root Mean Square (RMS) noise level for the PT within this work was deemed to be ±1°C. The RMS noise for the PT system was determined by measuring the standard deviation of the output voltage from the SiRs at the minimum DAQ unit acquisition time of 100 µs, post-conversion to temperature in °C. Various integration times were applied to the raw data during post-processing, corresponding to response times of 1ms, 10ms, 100ms, 500ms, 1s, and 2s. The mean noise across the dynamic temperature range as a function of integration time was used to inform the PT optimal acquisition time, ensuring noise fluctuations that were comparable to, or better than, the aforementioned thermocouples.

### 2.2. Absorptivity Measurement Methodology

The absorptivity of a material, α(λ), is a wavelength-dependent measure of the radiant flux that is absorbed and converted into internal energy, usually thermal energy, by that material [60]. It is linked to the principle of energy conservation, which also includes reflectivity, ρ(λ), and transmissivity, τ(λ) [61,62]. For opaque materials, such as the ones studied within this work, transmissivity can be considered negligible, meaning that all radiation can be treated as either being absorbed or reflected. Furthermore, the copper substrates, on which the MUTs are deposited, are opaque with a high density of states [63]. This, therefore, simplifies the relationship to Equation (2).

(2) α(λ)=1−ρ(λ)

This work utilises the temperature-dependent properties of MFG to detect the changes in temperature of two paints, and various mixing ratios of the two, when subject to irradiation from an SLS301 thermal source (Thorlabs Inc., Newton, NJ, USA) [48]. Absorptivity values can subsequently be inferred through empirical data and mathematical modelling. To achieve this, 12.5mm diameter samples were prepared from 0.1mm thick copper sheets, with one side coated with MFG and the other side coated with the MUT. For the samples, and the calibration disc, the MFG was mixed with Polyacrylamide (PAM) at a mass ratio of 10:1 (MFG:PAM). The MFG:PAM mixture was suspended in Isopropyl Alcohol (IPA), chosen for its high evaporation rate, at a concentration of 1 mL:1 g. Given that MFG is non-soluble in IPA, the solution was placed into an ultrasonic bath for 20min to aid with dispersion before being airbrushed onto the sample’s surface. The thickness of the MFG was measured to be approximately 0.01mm. Previous studies, such as Alden [26], explored various binders; however, PAM was chosen due to its availability and ability to withstand chain scission within the temperature ranges evaluated within this work [64].

Following the MFG coating, two samples were initially prepared for the absorptivity measurements, with one coated with a highly absorptive black paint ($\alpha_{vis}$ = 0.97), Black 3.0^®^ [65], and another with a contrasting, low-absorptivity white paint ($\alpha_{vis}=0.07$), Avian-B500^®^ [66]. Three subsequent paints were prepared using different ratios of Black 3.0^®^ and Avian-B500^®^ (75:25, 50:50, 25:75 White–Black) in order to investigate undefined intermediate absorptivities. The MUTs were applied to the copper surface using an airbrush and subsequently measured to have an approximate thickness of 0.01mm. Copper was chosen as the substrate material for the target samples due to its high thermal conductivity and specific heat capacity [67], helping to ensure a near-uniform temperature distribution throughout the material.

**Figure 4:**
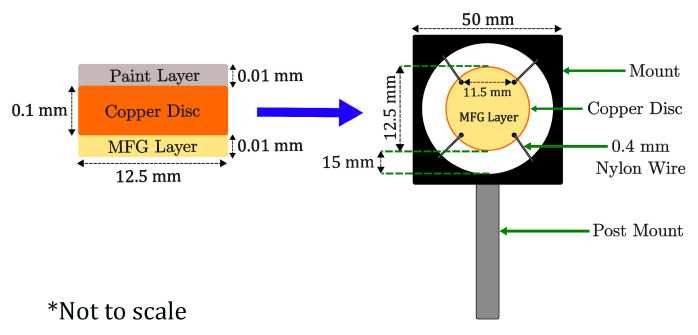
Sample schematic and mounting. The paint layer is represented in grey, the copper disc in orange and the MFG layer in yellow. The arrow is shown to indicate how the sample is subsequently mounted. * The figure is not to scale.

Four 1mm holes were created at equal distance close to the edge of the copper disc samples using a hole punch; these were used to suspend the disc in a mount using 0.4mm diameter nylon wire, as shown in Figure 4. In contrast to the copper, nylon was chosen due to its low thermal conductivity in order to maximise the thermal barrier between the mount and the copper disc [68]. If the mount was to be in thermal contact with the disc, energy absorbed from the thermal source would rapidly dissipate into the mount, therefore, introducing added uncertainty into the absorptivity measurements. The diameter of the disc was chosen to closely match that of the heat source’s beam diameter, ensuring the energy was evenly distributed across the disc.

**Figure 5:**
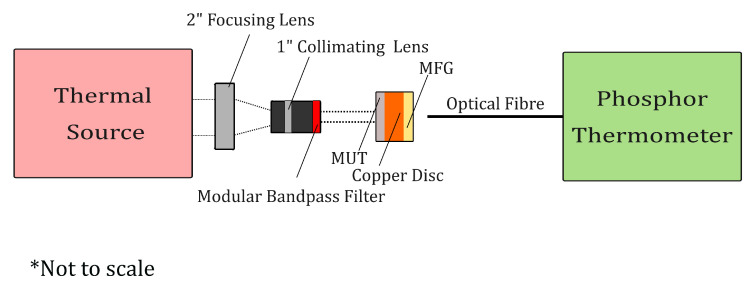
Absorptivity measurement setup schematic. The dashed lines represent the path of the incident thermal radiation. * The figure is not to scale.

Material absorptivity was evaluated using empirical heating data and mathematical modelling. To obtain the empirical data, the PT was aligned with the centre of the sample’s surface facing the MFG; the painted side (the MUT) was set to face the thermal source, which was optically aligned using N-BK7 plano-convex lenses. A schematic of the measurement setup is shown within Figure 5. The PT was set to capture the SiR output data for 30s before exposing the MUT side of the sample to the heat source, where 60s of heating data was subsequently captured; this was repeated three times for each MUT. A modular narrow bandpass filter was placed between the thermal source and the MUT when discrete wavelength absorptivity measurement was desired, as per Section 3.3.

The thermal model presented in Equation (3) describes the heating and cooling behaviour of a copper disc subjected to a steady-state energy source in a room-temperature environment. A key underlying assumption of this model is the lumped thermal capacity model, which simplifies the modelling [69]. The majority of the sample is made up of copper, which has an inherent high specific heat capacity $C_{c}$, with thin layers of the MUTs and MFG applied to either side. The specific heat capacity of the MUTs and MFG is not well characterised. Therefore, the lumped thermal model treats the entire sample as a single isolated object with a uniform temperature gradient, and the temperature differences within the sample are negligible. A better understanding of the specific heat capacity of the MUTs and MFG could inform a more accurate thermal model. However, characterisation of the specific heat capacity of these materials is beyond the scope of this work.

(3) $$\frac{dT(T)}{dt}=\frac{P_{in}}{mC_{c}}-kA\left(T\left(t\right)-T_{A}\right)$$

Each term represents a physical process influencing the disc’s thermal behaviour over time. $P_{in}$ represents the radiative power from the heat source, expressed as power per unit area, W/m^2^. The mass of the sample is denoted by *m*, given in kg. $C_{c}$ is the specific heat capacity of copper, with units J kg^−1^ K^−1^. *k* is the heat transfer coefficient, representing the gradient dT(t)/dT, which can be determined analytically or be inferred by fitting a linear regression model to the empirical data. The surface area of the sample is indicated by *A*, measured in m^2^. T(t) is the temperature of the copper disc, with units of *K* and $T_{A}$ corresponding to the ambient temperature at t=0, also in *K*. Substituting $k_{0}$ for kA and $\frac{P_{in}}{mC_{c}}$ for ρ, and integrating Equation (3), yields Equation (4), which describes the thermal behaviour of the copper samples during heating and cooling. A full derivation can be found within Appendix A.

(4) $$T{\left(t\right)}_{heat}=T_{A}+\frac{\rho }{k_{0}}\left(1-e^{-k_{0}t}\right)$$

The absorptivity, α(λ), can then be introduced as a product of the heating term, which yields Equation (5):

(5) $$T{\left(t\right)}_{\alpha }=T_{A}+\frac{\alpha (\lambda )\rho }{k_{0}}\left(1-e^{-k_{0}t}\right)$$

Equation (5) describes the heating behaviour of a given sample at a particular point in time, *t*. This equation was fitted to the empirical heating data and is used to infer the absorptivity of the MUTs. The absorptivity parameter, α(λ), was iteratively adjusted until the model was in agreement with the empirically measured heating data. Determination and analysis of the absorptivity parameter α(λ) are described within Section 3.2.

The total power output of the thermal source, after transmission and radiative losses, was found to be approximately 7 W between 0.35µm and 2.6µm. The impact of the optical filtering used to isolate discrete bands of Black 3.0^®^ contributed to a reduction in incident power; this reduction was accounted for within the model by utilising thermal and optical data from the manufacturer in conjunction with Equation (6). $P_{in}$ is the total radiative power and used within Equation (3), $P_{H}\left(\lambda \right)$ is the relative power from the heat source, F(λ) is the narrow-bandpass filter, and *A* is the area where the thermal energy is focused.

(6) $$P_{in}=P_{H}\left(\lambda \right)F\left(\lambda \right)A$$

**Figure 6:**
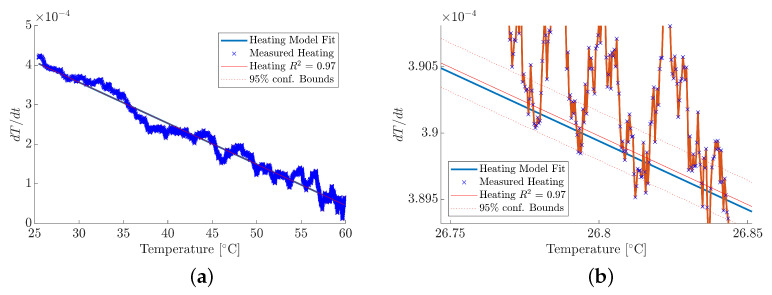
Representation of the methodology for obtaining absorptivity, α(λ), values. (**a**) MUT temperature gradient. (**b**) Magnified MUT temperature gradient.

To determine material absorptivity, α(λ), the empirical heating data captured by the PT was analysed through examination of the rate of change in temperature over a 60s period, defined by the 10–90% rise time. The thermal model described in Equation (5) begins with two unknown coefficients, the heat transfer coefficient, *k*, and the absorptivity value, α(λ). To determine *k*, a linear regression model was fitted to the empirical heating data, and the resultant gradient was extracted and substituted into Equation (5). Following this, the absorptivity parameter, α(λ), was systematically adjusted between 0 and 1 until the modelled temperature gradient was within the 95% confidence bounds of the linear regression fit, as shown in Figure 6. The measured temperature gradient for an MUT within the 10 to 90% of the maximum temperature is shown in Figure 6a, where a linear regression model is fitted to the empirical data. A magnified view is provided by Figure 6b, showing that the thermal model follows the overall empirical data trend, evaluated within the 95% confidence bounds. Furthermore, the experimental procedure was repeated three times for each MUT to ensure repeatability, where the quality of the fit is represented by the $R^{2}$ value. The median absorptivity of the three experiments was taken, and the minium and maximum measured values were used to inform the error within the measurement.

## 3. Results and Discussion

### 3.1. Instrument Characterisation

**Figure 7:**
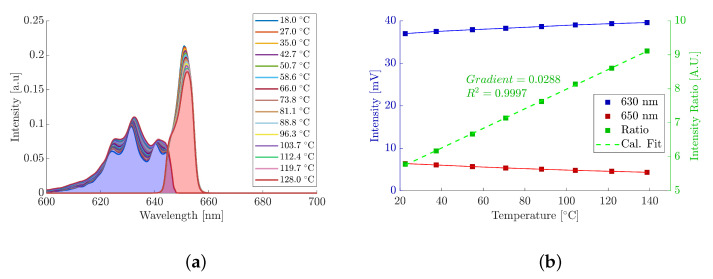
PT calibration results. (**a**) Isolated spectral emission intensity of MFG at different temperatures. (**b**) Calibration of the MFG phosphor emission as a function of temperature.

Before absorptivity measurements could take place, the PT was calibrated using the PT setup and procedure as outlined in Section 2.1. The area under each spectral emission curve, as measured by the spectrometer, is shown in Figure 7a and represents the total spectral power received by each of the SiRs as a function of temperature. The shorter spectral region (630nm peak) increases with an increase in temperature, whilst the longer spectral region (650nm peak) decreases with an increase in temperature. This measurement trend was replicated when the spectrometer was replaced by the SiRs, as shown in Figure 7b. The increase in temperature resulted in an increase in the 630nm SiR output voltage and a decrease in the 650nm SiR output voltage; this correlates with the spectral trend observed in Figure 7a. These output voltages changed as a function of temperature at a rate of 0.022 mV C^−1^ and −0.017 mV C^−1^ for the 630nm SiR and 650nm SiR, respectively. These trends indicate that the optical filters chosen for the use within the PT setup are producing the desired, reciprocal, temperature-dependent behaviours observed within the PT calibration model.

The ratio of the SiR outputs was subsequently taken and plotted together with the individual SiR outputs in Figure 7b. The ratio of the two SiR output voltages across the 20°C to 140°C temperature range exhibited a proportional and positive linear trend with increasing temperature in the form y=mx+c. In this configuration, the resolution of the PT is represented by the gradient, *m*, as shown in Figure 7b. A resolution of 0.0228 °C^−1^ was observed, which yields superior resolution to the previously mentioned single-channel SiR measurements. The scattering of the calibration points, around the fitted calibration line, is minimal with an $R^{2}>0.99$. This observed, strong correlation suggests that temperatures between the calibration points can be reliably interpolated. Furthermore, the linearity across the measured temperature range is comparable to similar calibrations reported using MFG [31]. The strong linear relationship enables straightforward conversion to temperature whilst also ensuring consistent temperature acquisition across the various samples.

**Figure 8:**
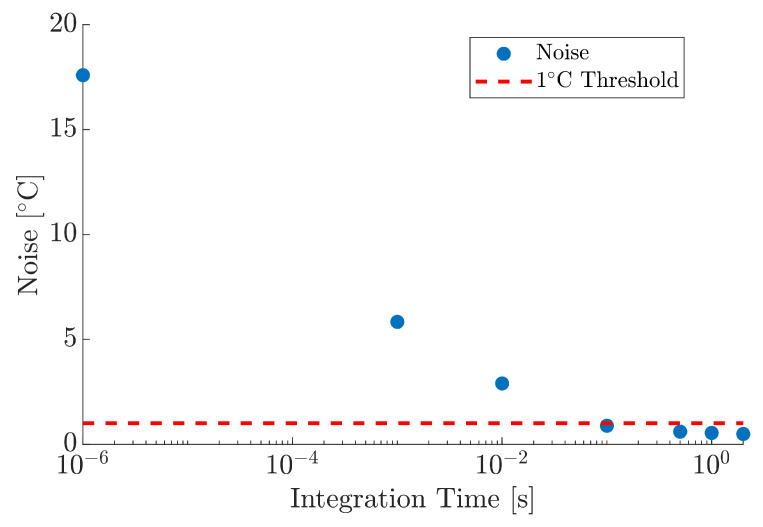
Mean temperature noise as a function of integration time.

The required data for the assessment of noise within the temperature measurement was acquired during the instrument’s calibration process; this data was subsequently assessed as a function of integration time, as shown in Figure 8. The results represent the mean noise taken across the full dynamic range of the calibrated instrument between 20°C and 140°C. The noise at the raw integration time of 100µs was measured to be 17.59°C, necessitating the need for increased integration. Observations of applying increasingly longer integration times reduced the magnitude of the mean noise across the entire temperature range; integration times of 1ms, 10ms, and 100ms reduced the mean noise to values of 5.83°C, 2.89°C, and 0.88°C, respectively. The mean noise was reduced further to 0.59°C, 0.54°C and 0.48°C when integration time was increased to 500ms, 1s, and 2s, respectively. The 1s integration time was subsequently used throughout this work to ensure SNR remained high, random noise errors were reduced, and comparable sampling speeds of typical thermocouples were maintained [59].

**Figure 9:**
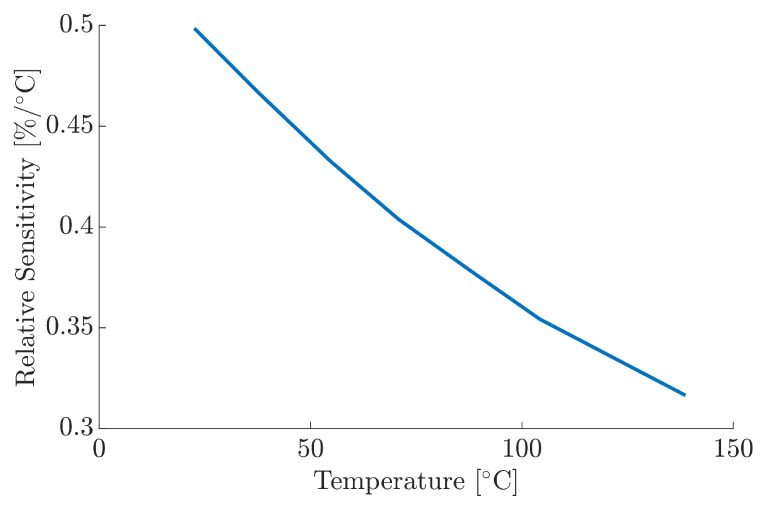
Relative sensitivity, $S_{r}$, of the MFG ratio signal as a function of temperature.

There are several mechanisms that perpetuate higher levels of noise within radiometry, most notably photon noise, which arise from the statistical fluctuations of photons emitted by the MFG. This is an unavoidable constraint, especially at low signal levels, such as the case within this work due to the use of optical filtering and beam splitters. The implementation of higher sensitivity photodetectors could alleviate some of the sensitivity limitations associated with photodiodes. Further potential explanations for the impact of noise relates to the temperature of the hot plate. When the hot plate heats up, the temperature gradient increases due to the ambient temperature of the surrounding air, resulting in additional thermal noise as the hot plate attempts to stabilise itself. The relative sensitivity $S_{r}\;\left[1/R\left(dR/dT\right)\right]$ for MFG, as shown in Figure 9, shows a reduction as a function of temperature; although this is not directly related to the noise, the diminishing sensitivity, as a function of increase temperature, influences the signal within the SNR and, therefore, measurement uncertainty.

### 3.2. Absorptivity Measurements

**Figure 10:**
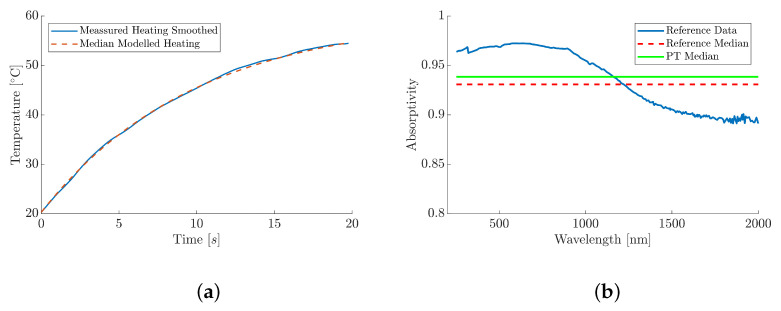
Absorptivity results for the median absorptivity of Black 3.0^®^. (**a**) Median empirical and modelled temperature curve for Black 3.0^®^. (**b**) Absorptivity data for Black 3.0^®^ extracted from [19].

Having characterised and calibrated the PT, it was subsequently used to measure the absorptivity of two contrasting paints, along with various mixtures of the two paints. Firstly, the absorptivity of the MUT, Black 3.0^®^, was obtained by heating it with an incident radiation between 0.2µm and 2µm. The absorptivity was subsequently calculated and compared against a reference extracted from published spectral absorptivity data [19]. The MUTs were heated by the thermal source from room temperature, and the temperature data was analysed within the 10% to 90% range of the heating curve; for the black paint, this corresponded to a median time of 19.6s. The median empirical and modelled temperature curve, as a function of time, is plotted within Figure 10a. Three measurements, as previously described in Section 2.2, were recorded using the PT setup, and the thermal model was subsequently used to obtain a median absorptivity value. The absorptivity values for each measurement were determined, and the results are shown in Table 1.

The median temperature increase for the black paint was found to be 34.1°C within the 10% to 90% range. From this, the median absorptivity was subsequently calculated to be 0.94, within a range of 0.0081; this is plotted alongside the reference data within Figure 10b. The thermal model showed a high fit quality to the empirical heating data, with an $R^{2}$ greater than 0.95; this high $R^{2}$ value confirms a good agreement between the empirical and modelled results, validating the model’s reliability for predicting thermal behaviour.

The reference data, shown in Figure 10b, indicates that Black 3.0^®^ has an absorptivity of approximately 0.97 within the UV to visible spectrum (0.2–0.75 µm), which is the value quoted by the manufacture [65]. The absorptivity reduces to less than 0.9 as the wavelength increases through to the Near-Infrared (NIR) and Short-Wave Infrared (SWIR) spectral regions (0.75 to 2µm). The median absorptivity value from the reference data, across the entire 0.2µm to 2µm spectral region, was calculated to be 0.93; this value was subsequently used to evaluate the validity of the PT and the mathematical modelling and is shown by the dashed line within Figure 10b. A percentage difference of 0.8% was observed between the reference data and the PT method; this small relative difference suggests that PT can be a viable method for determining material absorptivity.

**Figure 11:**
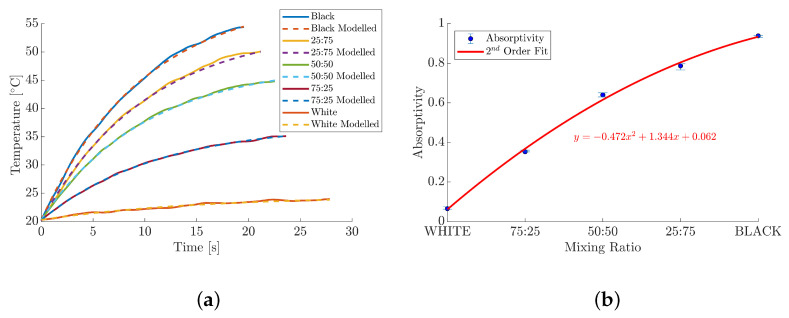
Median absorptivity and heating results for MUTs across the 0.2µm to 2µm spectral region. (**a**) Median empirical and modelled heating curves for all MUTs. (**b**) Absorptivity as a function of paint mixing ratios White–Black.

To further assess the PT technique for absorptivity measurements, the white paint, Avian-B500^®^, and the three intermediate mixtures were used to provide contrast to the highly absorptive Black 3.0^®^. The median and modelled heating curves for each MUT are presented in Figure 11a. The median time taken for a given MUT to go between the 10% and 90% of its maximum temperature was reduced as a function of increasing black paint percentage at a decelerating rate of 28.7s, 23.6s, 22.5s, 21.81s, and 19.6s, respectively. The median differences in temperature rise, from the white paint to the black paint, were 3.6°C, 14.9°C, 24.5°C, 29.9°C, and 34.1°C, respectively.

The absorptivity of each MUT was subsequently calculated from the heating measurements and presented within Figure 11b, where the error bars represent the range between the absorptivities of the repeat experiments. The individual results for each experiment are included within Table 2. The white paint was found to yield a median absorptivity of 0.065 within a range of 0.0127, which is consistent with the manufacturer’s specification of α(λ)<0.07 over a wavelength range of 250nm to 1300nm. Although the exact absorptivity of the three paint ratios was unknown, it was hypothesised that the values would be a function of their respective paint ratio and, therefore, fall between the known values of the pure paints. The 75:25, 50:50, and 25:75 ratios (White–Black) were measured to have median absorptivities of 0.35, 0.64, and 0.79, within a range of 0.0092, 0.0212, and 0.0309, respectively. These results show an expected, but clear, trend: increasing the relative mass content of Black 3.0^®^ leads to an increase in the overall absorptivity of that MUT. A second-order polynomial was subsequently fitted to the absorptivity data, yielding a strong correlation with $R^{2}>0.95$. The non-linearity in the absorptivity results, as a function paint mixing ratio, suggests that the absorptive properties of the black paint pigment, and pigment density, dominate the optical characteristics of the MUTs.

A clear relationship between the MUTs absorptivity, its maximum temperature, and the time it takes to reach that temperature is shown within Figure 11. Both the relative temperature increase and corresponding absorptivity value of the MUTs decrease as a function of increasing black paint percentage; this trend is illustrated by the polynomial fit within Figure 11b. The trend reveals a characteristic inverse relationship between heating time and the maximum temperature: materials that exhibit a high α(λ) and a low ρ(λ) efficiently absorb radiant energy, leading to a rapid rise in internal energy and temperature. Conversely, materials that reflect most incident energy show a minimal temperature increase over a longer duration in time.

Black 3.0^®^ consists of a high-density and proprietary pigment that is naturally very matte [65]. Additionally, its formula includes transparent mattifiers that flatten the surface and reduce stray light at a microscopic level; this could explain the non-linear increase in the temperature rise from room temperature. Therefore, when greater percentages of black paint are added to the mixtures, there is an increase in pigment density, the mixture reaching its maximum temperature at an accelerate rate. It should also be noted that the reduction in temperature rise for the lower absorptivity MUTs is directly related to the magnitude of the signal measured by the PT over time, therefore, resulting in a reduction in the SNR. Despite this, the consistency of the results demonstrates good repeatability of the PT measurements across the absorptivity range and acts as a good indication that it can predictably measure known and unknown absorptivity values.

### 3.3. Narrow-Band Absorptivity Measurement

To enable an assessment of the performance of the PT absorptivity measurement technique at discrete wavelengths, the wavelength-dependent absorptivity of the Black 3.0^®^ sample used within Section 3.2 was investigated. To achieve this, two narrow bandpass optical filters, each with full width half maximums (FWHMs) of 40nm, were positioned between the thermal source and the MUT for their respective tests. One filter was within the visible wavelength spectral range at 600nm, and the other was within the SWIR spectral range at 1500nm. These specific narrow spectral regions were chosen to enable a clear demonstration of the PT technique for the measurement of material absorptivity as a function of wavelength. The results were compared to the reference data obtained from previously established work [19].

**Figure 12:**
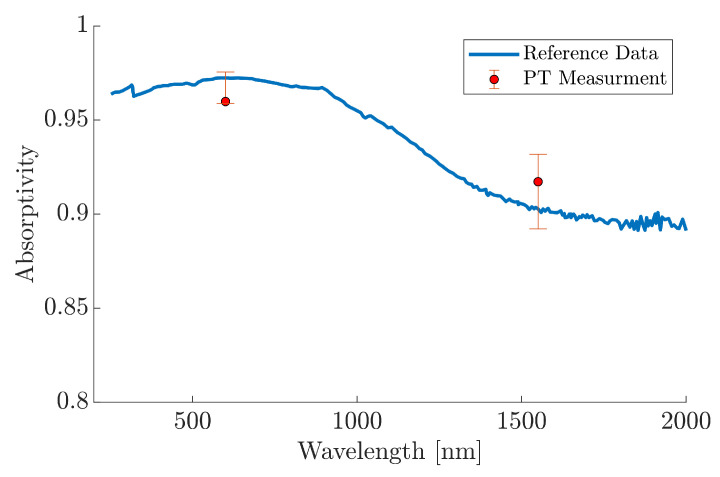
PT absorptivity measurements at discrete wavelengths. Reference data extracted from [19].

The PT captured three sets of heating data for each of the narrow bandpass optical filters. The median absorptivity values were found to be 0.9598 and 0.9172, within a range of 0.0168 and 0.0396 at 600nm and 1500nm, respectively. These values are plotted in Figure 12 alongside the experimental absorptivity data extracted from [19]. The error bars represent the minimum and maximum measured range for both of the wavelength-dependant tests. The reference data, represented by the blue data within Figure 12, clearly pass through the both sets of error bars associated with each of the PT measurements. The percentage difference in the median absorptivity measurement relative to the reference data at specific wavelength was calculated to be 1.3% and 1.6% for the 600nm and 1500nm, respectively. This result provides evidence for the PTs capability of resolving specific, narrow-band absorptivity. However, a significant reduction in temperature rise over the heating period was observed at 1550nm, with the median measurement changing by just 1.1°C compared to 10.3°C at 600nm. The reduced temperature rise within these measurements can be attributed to the optical filters, which significantly lowered the proportion of incident radiation upon the MUT. Consequently, this reduction in the temperature rise resulted in a lower SNR, which is represented by the noticeably larger error bars at 1550nm compared to those at 600nm. With a lower noise system, these error bars could be reduced and the median absorptivity converge towards the established data. Uncertainties, such as variations within the layer thickness of the composite sample, could also contribute to an overall reduction in SNR. The samples comprise of composite layers, which are independently effected by the incident radiation. The incident radiation could pass through two or more sufficiently thin layers; this requires consideration for the discrepancy within the negative and positive shifting at the different measured wavelengths.

The decrease in spectral intensity at 1550nm is defined by Planck’s law of EMR, with the thermal source manufacturer quoting a steady-state temperature of 3400K. Whilst perfect blackbody emitters are not physically possible, the thermal source loosely follows the analytical solution to Planck’s law; this is supported with empirical spectral data provided by the manufacturer [48]. At this temperature, there is a reduction in the spectral intensity emitted within the NIR and SWIR spectral regions. It is, therefore, hypothesised that the differences in temperature rise caused by optical filtering, $\Delta T_{F}$, may not be directly proportional to the decrease in incident power $P_{in}$. This non-linearity between power and temperature is typically governed by the combined laws of convection and radiation heat transfer [70]. The consequence of this is that the difference in $\Delta T_{F}$ between any two discrete power values becomes more pronounced at the lower end of the power scale. Hence, the temperature rise for the 600nm region is significantly higher than the 1550nm region despite the marginal reduction in spectral power. The reduction in the spectral power, caused the by optical filtering, was compensated for within the model using the optical filter data, F(λ), within Equation (6), and the resulting power values are presented within Table 3.

To overcome the inherent challenges associated with the thermal source’s relatively low incident power, alternative heating methods could be considered. Higher power monochromatic light sources, such as lasers, could be used to heat the MUTs. Such systems would provide a higher, concentrated power density at distinct wavelengths, improving the SNR, and enable precise mapping of a material’s absorptivity at critical application-specific wavelengths. Furthermore, a better understanding of the specific heat capacity of each of the MUTs and MFG could improve the accuracy of the thermal model. The PT method would be particularly valuable for industrial applications, such as SLM, where wavelength-dependent material absorptivity at a specific wavelength directly impacts both energy efficiency and the quality of production.

## 4. Conclusions

This study introduced a low-cost, non-contact, PT calorimetric technique for determining the optical absorptivity of materials. By combining PT with mathematical modelling, the system demonstrated the ability to resolve absorptivity values of both known and unknown materials, including for measurement scenarios that were signal limited. The technique was validated by measuring the absorptivity of Black 3.0^®^, resulting in a 0.8% discrepancy from reference data. Further experiments with a low-absorptivity paint, Avian-B500^®^, and various intermediate mixtures of the two paints confirmed the system’s capabilities across a contrasting range of absorptivity values. The system was then shown to be capable of measuring absorptivity for narrow-band wavelengths, with results at 600nm and 1500nm aligning closely with established data. The technique circumvents the limitations of traditional radiometric and calorimetric methods, which are subject to emissivity uncertainty or the need for optimal thermal contact, respectively. This PT technique could enable low-cost, non-contact, and portable absorptivity measurement systems, removing the need for complex and expensive test setups. This technique has the potential to be utilised across various engineering fields that benefit from an understanding of wavelength-specific material absorptivity.

## Figures and Tables

**Table 1:** Results for unfiltered Black 3.0^®^ absorptivity across a wavelength range of 0.2µm to 2µm.

Power [*W*]	Test #	Absorptivity α(λ)	Median α(λ)
	1	0.9385	
7	2	0.9312	0.9385
	3	0.9393	

**Table 2:** Absorptivity results for various paint ratios across a wavelength range of 0.2µm to 2µm.

MUT	Power [W]	Test #	Absorptivity α(λ)	Median α(λ)
		1	0.0620	
White	7	2	0.0651	0.0651
		3	0.0747	
		1	0.3609	
25:75	7	2	0.3517	0.3528
		3	0.3528	
		1	0.6509	
50:50	7	2	0.6297	0.6401
		3	0.6401	
		1	0.7662	
75:25	7	2	0.7971	0.7868
		3	0.7868	
		1	0.9385	
Black	7	2	0.9312	0.9385
		3	0.9393	

**Table 3:** Absorptivity results for Black 3.0^®^ using narrow bandpass filters with a FWHM =40nm.

Filter Wavelength [nm]	Power [W]	Test #	Absorptivity α(λ)	Median α(λ)
		1	0.9755	
600–40	0.178	2	0.9598	0.9598
		3	0.9587	
		1	0.9318	
1550–40	0.106	2	0.9172	0.9172
		3	0.8922	

## Data Availability

The original contributions presented in this study are included in the article. Further inquiries can be directed to the corresponding authors.

## Abbreviations

The following abbreviations are used in this manuscript:

EMR	Electromagnetic Radiation
FWHM	Full-Width Half-Maximum
IR	Infrared
IPA	Isopropyl Alcohol
IRT	Infrared Radiation Thermometer
LED	Light-Emitting Diode
LWIR	Long-Wave Infrared
MFG	Manganese-activated Magnesium Fluorogermanate
MUT	Material Under Test
MRI	Magnetic Resonance Imaging
NIR	Near-Infrared
PD	Photodiode
PL	Photoluminecent
PT	Phosphor Thermometry
RMS	Root Mean Square
SiPD	Silicon Photodiode
SiR	Silicon Photodiode-based Radiometer
SLM	Selective Laser Melting
SNR	Signal-to-Noise Ratio
SWIR	Short-Wave Infrared
TP	Thermographic Phosphor
TIA	Transimpedance Amplifier
UV	Ultraviolet
VIS	Visible
VLWIR	Very-Long-Wave Infrared

## Appendix A. Derivation of Heating and Cooling Terms for Mathematical Analysis

The thermal model shown within Equation (A1) allows for descriptions of both the heating and cooling behaviour of the samples to be obtained. Only the heating term was used within this work; however, both derivations are shown here for completeness.

(A1) $$\frac{dT(T)}{dt}=\frac{P_{in}}{mC_{c}}-kA\left(T\left(t\right)-T_{A}\right)$$

where

$$\begin{array}{cc} P_{in} & =\mathrm{Radiative}\;\mathrm{power}\;\mathrm{from}\;\mathrm{the}\;\mathrm{thermal}\;\mathrm{source}\;\left(\frac{\mathrm{Power}}{\mathrm{Area}}\right)\;\left[W/m^{2}\right].\\ m & =\mathrm{Mass}\;\mathrm{of}\;\mathrm{the}\;\mathrm{copper}\;\mathrm{disc}\;[\mathrm{kg}].\\ C_{c} & =\mathrm{Specific}\;\mathrm{heat}\;\mathrm{capacity}\;\mathrm{of}\;\mathrm{copper}\;[J\;{\mathrm{kg}}^{-1}\;K^{-1}].\\ k & =\mathrm{Heat}\;\mathrm{transfer}\;\mathrm{coefficient}\;(\mathrm{Gradient}).\\ A & =\mathrm{Surface}\;\mathrm{area}\;\mathrm{of}\;\mathrm{the}\;\mathrm{copper}\;\mathrm{disc}\;[m^{2}].\\ T(t) & =\mathrm{Temperature}\;\mathrm{of}\;\mathrm{the}\;\mathrm{disc}\;\mathrm{at}\;\mathrm{time}\;t\;[K].\\ T_{A} & =\mathrm{Ambient}\;\mathrm{temperature}\;[K]. \end{array}$$

Integrating Equation (A1) gives

(A2) $$\int \frac{1}{\rho -k_{0}\left(T\left(t\right)-T_{A}\right)}dT=\int dt$$

(A3) $$-\frac{1}{k_{0}}\ln\left(\rho -k_{0}\left(T\left(t\right)-T_{A}\right)\right)=t+c$$

where the constant $\rho =\frac{P_{in}}{mC_{c}}$, and $k_{0}=kA$. Rearranging for, T(t), yields

(A4) $$T\left(t\right)=T_{A}+\frac{\rho -\epsilon e^{k_{0}t}}{k_{0}}$$

The unknown constant, ϵ, has two unique solutions, both of which are dependent on the current phase, heating, $\epsilon_{H}$, and cooling, $\epsilon_{C}$. First, the heating constant, $\epsilon_{H}$, was determined at t=0. Equation (A4) during the heating phase becomes

(A5) $$T\left(0\right)=T_{A}+\frac{\rho -\epsilon_{H}}{k_{0}}$$

At t=0, $T\left(0\right)=T_{A}$. Therefore, rearranging Equation (A5) for $\epsilon_{H}$ yields

(A6) $$\epsilon_{H}=\rho$$

Substituting, $\epsilon_{H}$, into Equation (A4) yields Equation (A7), which is the heat equation used within this work:

(A7) $$T{\left(t\right)}_{heat}=T_{A}+\frac{\rho }{k_{0}}\left(1-e^{-k_{0}t}\right)$$

Finally, the cooling constant, $\epsilon_{C}$, was evaluated at the end of the heating phase when the thermal source is switched off, with T(t) starting at the peak of the heating curve. Therefore, during the cooling phase, $T\left(0\right)=T_{max}$. Setting the input power, $P_{in}=0$, subsequently results in ρ=0. Substituting these conditions into Equation (A4) gives

(A8) $$T_{max}=T_{A}-\frac{\epsilon_{C}}{k_{0}}$$

Rearranging Equation (A8) for $\epsilon_{C}$ yields

(A9) $$\epsilon_{C}=k_{0}\left(T_{A}-T_{max}\right)$$

Substituting $\epsilon_{C}$ into Equation (A4) yields

(A10) $$T{\left(t\right)}_{cool}=T_{A}-\left(T_{A}-T_{max}\right)e^{-k_{0}t}$$

The gradient, *k*, for both heating and cooling terms can be derived from these equations and yields a good approximation. However, applying a linear fit to the empirical heating data, $\frac{dT(t)}{dt}$, resulted in a more robust and applicable approach to this work.

## Appendix B. Bill of Optical Materials Used in PT Design Setup

**Table A1:** Bill of materials for the key components used in the instrument design.

Component	Part Number	Manufacturer
405nm LED	LED405L	Thorlabs Inc.
Glass Lens (f=50mm)	LA1131-ML	Thorlabs Inc.
50:50 Beam Spillter	CCM1-BS013/M	Thorlabs Inc.
Optical Fibre (1500µm Core)	M93L01	Thorlabs Inc.
550nm Longpass Filter	FELH0550	Thorlabs Inc.
650nm Shortpass Filter	FESH0650	Thorlabs Inc.
658nm Notch filter	NF658-26	Thorlabs Inc.
650nm Bandpass Filter	FBH650-40	Thorlabs Inc.
Silicon Photodiode	S5971	Hamamatsu Inc.
$Mg_{3.5}FGeO_{5}$:Mn(MFG)	EQD25/F-U1	Phosphor Technology Inc.
